# Vaginal birth after two cesarean sections (VBAC-2) under a standardized protocol: success rates, safety, and cesarean after spontaneous labor as an alternative

**DOI:** 10.1186/s12884-026-08905-9

**Published:** 2026-03-12

**Authors:** Anna Elisabeth Hentrich, Dörthe Brüggmann, Juliane Bresgen, Samira Catharina Hoock, Eileen Deuster, Frank Louwen, Lukas Jennewein

**Affiliations:** Department of Obstetrics and Perinatal Medicine, University Hospital, Goethe University Frankfurt, Theodor-Stern-Kai 7, Frankfurt am, 60590 Germany

**Keywords:** Vaginal birth after cesarean, VBAC-2, Cesarean section, Uterine rupture, Maternal outcomes, Neonatal outcomes

## Abstract

**Background:**

An increasing number of women with two previous cesarean sections are seeking personalized delivery options. However, evidence on the safety of a vaginal birth after two cesarean sections (VBAC-2) under standardized, non-induction protocols remains limited. This study aimed (1) to assess success rates and safety of vaginal birth after two cesarean sections (VBAC-2) under a standardized non-induction protocol and (2) to compare maternal and neonatal outcomes between elective repeat cesarean (ERCS-2) and cesarean after spontaneous labor onset (CSAOL-2).

**Methods:**

We conducted a retrospective single-center cohort study including women with singleton pregnancies between 36 + 0 and 41 + 6 weeks and two prior low-transverse cesareans (2014–2025). Counseling followed a predefined protocol. VBAC-2 was only offered with spontaneous labor onset. Primary outcomes were VBAC-2 success (intent-to-treat) and intraoperative uterine rupture or dehiscence. Secondary outcomes were maternal morbidity, blood loss, and neonatal outcomes.

**Results:**

Of 390 women, 107 intended VBAC-2. 33 achieved vaginal birth (16 spontaneous, 17 forceps), corresponding to a success rate of 30.8% (intent-to-treat). No complete rupture occurred among VBAC-2 attempts. Across the cohort, the overall complete rupture rate was 1.0%, and dehiscence occurred in about 8.2%. Mean blood loss was significantly lower after successful VBAC-2 compared with repeat cesarean (371 ml vs. 473 ml; *p* < 0.001). CSAOL-2 showed no excess maternal or neonatal morbidity compared with ERCS-2 although a non-significant numerical increase in complete uterine rupture was observed.

**Conclusion:**

VBAC‑2 is feasible under strict selection and standardized non‑induction conditions, but success rates are modest and instrumental delivery is frequent. In this underpowered cohort, no complete uterine ruptures occurred among VBAC‑2 attempts. CSAOL-2 did not show statistically significant differences in maternal or neonatal morbidity compared with ERCS-2; however, these findings do not provide a sufficient medical rationale to routinely wait for the onset of labor solely in order to perform a cesarean delivery. This approach may be reserved for women who remain undecided about mode of delivery despite comprehensive counseling.

**Supplementary Information:**

The online version contains supplementary material available at 10.1186/s12884-026-08905-9.

## Background

Global cesarean section rates continue to rise, with projections suggesting nearly one in three births worldwide may occur by cesarean by 2030 [1]. Consequently, an increasing number of women present with pregnancies after two prior cesarean deliveries. Management of delivery in this setting remains challenging: while guidelines from the American College of Obstetricians and Gynecologists (ACOG) and the German Society of Gynecology and Obstetrics do not contraindicate a trial of labor after two cesareans (VBAC-2) in the absence of other risk factors [2, 3], concerns about uterine rupture and medicolegal implications often lead to planned repeat cesarean.

In addition to maternal safety considerations, elective repeat cesarean performed before labor onset is associated with increased neonatal respiratory morbidity and adjustment disorders [2, 4]. Allowing spontaneous labor before cesarean (CSAOL-2) may mitigate these risks, yet systematic evidence is limited.

To support informed counseling, robust real-world data under standardized protocols are needed. The primary objective of this study was to determine the success rate and uterine rupture/dehiscence risk of VBAC-2 under a standardized non-induction protocol in women with two previous cesarean sections. Secondary objectives were to compare maternal and neonatal outcomes between elective repeat cesarean (ERCS-2) and cesarean after spontaneous labor onset (CSAOL-2) in this cohort.

## Materials and methods

### Study design and setting

We conducted this monocentric retrospective cohort study from 2014 to 2025 at a state-level perinatal center in Hessen, Germany. All patients with a history of two previous cesarean sections who delivered at our center during the study period were eligible. Additional inclusion criteria were a singleton pregnancy and a gestational age between 36 + 0 and 41 + 6 weeks. STROBE guidelines were followed.

### Participants

#### Inclusion criteria

- Singleton pregnancy.
- Gestational age 36 + 0 to 41 + 6 weeks.
- Two prior low-transverse cesarean sections.

#### Exclusion criteria

- Uterine incision other than low transverse.
- Contraindication to vaginal delivery: placenta previa, fetal presentation incompatible with vaginal delivery.
- A history of uterine rupture or uterine dehiscence in a previous cesarean section.
- Markedly thinned lower uterine segments or sonographic findings suggestive of preexisting myometrial dehiscence.
- Fetal-maternal disproportion (clinical examination or previously identified in intraoperative measurements.
- Multiple pregnancy.
- Major fetal anomaly.
- Fetal macrosomia with > 4000 g.
- Poorly regulated diabetes.
- Maternal disease like preeclampsia.

If patients did not fulfill these criteria, they were excluded from the VBAC-2 group and from the statistical analysis of cesarean section outcomes for comparison purposes.

The study was approved by the institutional review board of Goethe University Frankfurt (IRB 2021‑325) and conducted in accordance with the Declaration of Helsinki. As this was a retrospective analysis based on anonymized data collected during standard clinical care, the ethics committee waived the requirement for additional informed consent. Data were extracted from the statewide “Perinatalerhebung Hessen” database and the hospital’s electronic medical records.

### Exposure groups

#### Intention-based groups

(1) VBAC-2: vaginal birth after two cesareans (2) ERCS-2: elective repeat cesarean before labor (3) CSAOL-2: cesarean after spontaneous labor onset.

#### Outcome-based groups

(1) VBAC-2: vaginal birth after two cesareans (spontaneous or forceps-assisted) (2) ERCS-2: elective repeat cesarean before labor (3) CSAOL-2: cesarean after spontaneous labor onset (a) without vaginal intention, and (b) failed TOLAC-2.

### Counseling and management

All eligible patients received standardized counseling between 35 + 0 and 36 + 6 weeks of gestation. Counseling included verbal and written information using an official institutional consent form. This form covered options for mode of delivery, including spontaneous vaginal delivery, operative vaginal birth (e.g., forceps), planned or unplanned cesarean section, and associated risks such as uterine rupture, postpartum hemorrhage, and birth injuries. Special emphasis was placed on risks in women with previous cesarean sections.

Ultrasound evaluation of the uterine scar was performed to assess the risk of rupture, using a lower uterine segment myometrial thickness threshold of 2 mm, as recommended by the ISUOG guidelines [5]. Patients with markedly thinned lower uterine segments or sonographic findings suggestive of preexisting myometrial dehiscence were counseled against attempting VBAC-2 and were managed with elective repeat cesarean delivery.

Structured decision aids or predictive tools (e.g., VBAC calculators) were not formally applied. Instead, counseling was individualized based on obstetric history. Prior indications for cesarean section were reviewed in detail with the patient to estimate the likelihood of successful vaginal delivery.

Individualized counseling considered prior cesarean indications and current risk factors. Cephalopelvic disproportion (CPD) was suspected based on clinical pelvic exam, failure of presenting part engagement near term, or fetal biometry suggesting macrosomia (≥ 4000 g, exclusion criterion). Patients with prior arrest of dilation/descent were counseled conservatively but CSAOL-2 offered per shared decision-making.

Shared decision-making was prioritized, incorporating the patient’s preferences, risk factors, and the clinical assessment. A vaginal birth after two cesareans (VBAC-2) was only offered in cases of spontaneous onset of labor. Labor induction was not performed. Patients were also informed about the option of a planned cesarean section after spontaneous labor onset (planned CSAOL-2), providing an intermediate option between elective cesarean section and VBAC-2. This counseling process was maintained throughout the entire study period. The exclusion criteria remained consistent over the 10-year timeframe. Only the written consent form was introduced five years ago.

Delivery was supervised by a team consisting of a board-certified obstetrician, a resident physician, and a midwife.

In cases where patients opted for VBAC-2 and spontaneous labor did not occur by the due date; shared decision-making was applied. Patients were counseled regarding expectant management and the duration they wished to continue the pregnancy in the absence of labor. From our side, a repeat cesarean delivery was recommended seven days after the estimated due date if labor had not started spontaneously.

In cases of prelabor rupture of membranes, expectant management was followed according to our internal hospital protocol for up to 48 h, which corresponds to the time frame during which labor induction would otherwise be initiated.

### Outcomes

Primary outcomes were:

1. Intent-to-treat: Success rate of vaginal birth after two cesarean sections (VBAC-2) with vaginal intention.
2. Rate of complete rupture and dehiscence forms.

For uterine rupture assessment, all surgical and delivery reports were reviewed for evidence of full-thickness dehiscence (complete rupture) or partial separation of the uterine muscle stabilized by surrounding structures (uterine dehiscence). Clinical signs such as scar pain or pathological cardiotocography were also considered.

Secondary outcomes included maternal and neonatal morbidity and mortality. Maternal complications analyzed were blood loss (in mL), blood transfusion, uterine atony, urinary bladder injury, hysterectomy, third- or fourth-degree perineal trauma, and surgical wound complications. Neonatal outcomes included arterial cord blood pH, respiratory distress syndrome, intubation, NICU admission, 5-minute APGAR < 4, neonatal infection, and postnatal adjustment disorder. The age at discharge (in days) was also recorded.

### Statistical analyses

Statistical analyses were conducted using JMP version 14.0 (SAS Institute, Cary, NC, USA). Continuous variables were assessed for normality using Kolmogorov-Smirnov test. Primary outcomes (VBAC-2 success rate, uterine rupture/dehiscence) were analyzed using bivariate methods (Student’s t-test, Mann-Whitney U test, Fisher’s exact test). Exploratory nominal logistic regression (Table 7) examined factors associated with vaginal delivery intention (dependent variable: VBAC-2 intention vs. elective cesarean intention), using selected outcomes (pHa < 7.15, NICU admission, postpartum hemorrhage, uterine rupture) as independent variables. Due to low event rates, regression results are descriptive only and not interpreted causally.

All tests were two-sided and a p-value of less than 0.05 was considered statistically significant.

## Results

### Cohort characteristics

The study comprised a total of 390 participants. The mean age of the subjects was 39.7 years, and the mean BMI was 27.0 kg/m². As demonstrated in Table 1, baseline characteristics exhibited no significant disparities between the groups, except for gestational age at delivery, which was slightly higher among women with VBAC-2 intention.

**Table 1:** Characteristics, outcome related groups, VBAC-2 = vaginal birth after two cesarean sections, RCS-2 includes elective repeat cesarean sections before labor (ERCS-2, *n* = 237) and cesarean sections after spontaneous labor onset (CSAOL-2, *n* = 116). Continuous variables were compared using Student’s *t*-test or Mann–Whitney *U* test, as appropriate; categorical variables using Fisher’s exact test

Characteristics	total *n* = 390	VBAC-2 *n* = 37	Cesarean section *n* = 353	*p*-value
Age (years, mean ± SD)	39.7 ± 6.1	40.8 ± 5.2	39.6 ± 6.2	0.306
BMI (kg/m², mean ± SD)	27 ± 6.0	25.2 ± 3.7	27.2 ± 6.2	0.085
Parity	3.06	3.08	3.06	0.860
Duration of pregnancy (weeks, mean ± SD)	38.8 ± 1.2	39.6 ± 1.4	38.7 ± 1.2	< 0.001
Gestational diabetes (n, %)	54 (14.1)	3 (8.1)	52 (14.7)	0.271
Vaginal birth in history (n, %)	24 (6.2)	4 (10.8)	20 (5.7)	0.215
Fetal birth weight (grams, mean ± SD)	3428.9 ± 496	3567 ± 491	3415 ± 495	0.236

### Delivery intention and VBAC-2 success rates

A total of 107 women had a documented VBAC-2 intention. Of these, 81 (75.7%) experienced spontaneous labor and therefore underwent TOLAC‑2 under our non‑induction protocol. Among women undergoing TOLAC-2, 33 achieved VBAC-2, corresponding to an intent-to-treat success rate of 30.8% (33/107).

**Fig. 1:**
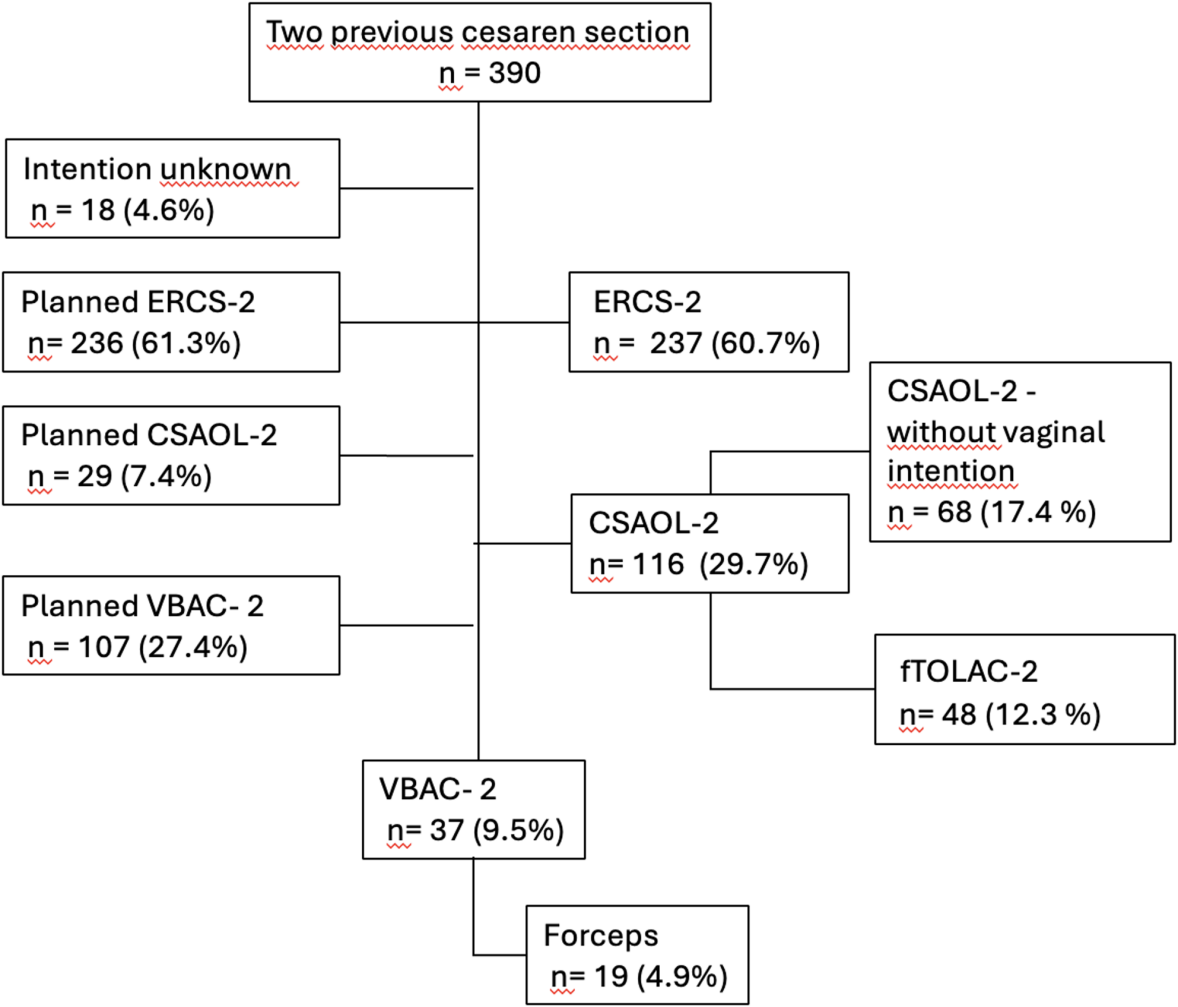
Flowchart of patient inclusion, delivery intention, and actual outcomes

In addition, 2 further women had an unclear documented delivery intention and two no documented vaginal delivery intention, but also achieved vaginal birth, resulting in a total of 37 vaginal births after two cesarean sections. Of these 37 vaginal births, 18 were spontaneous and 19 required forceps-assistance. The overall VBAC-2 rate in the cohort was 9.5% (37/390). Of the remaining patients, 237 underwent ERCS-2 and 116 underwent CSAOL-2. The flowchart is presented in Fig. 1.

### Intended and actual mode of birth

The delivery intentions and actual outcomes are summarized in Table 2 and illustrated in Fig. 1. In a study of 390 women, a total of 107 patients attempted a trial of labor for vaginal birth after two cesarean sections (VBAC-2) according to our standardized institutional protocol, of whom 33 achieved vaginal birth (30.8%), while 48 required an intrapartum cesarean section (failed TOLAC-2). Of the 107 women who initially intended a vaginal birth, 26 ultimately underwent an elective repeat cesarean section because spontaneous labor did not occur and labor induction was not offered in our protocol, for example in cases of prolonged pregnancy or pre-labor rupture of membranes. Of the 236 women who were planning to undergo ERCS-2, all underwent cesarean section prior to the onset of labor.

42 underwent cesarean section after spontaneous labor onset, without vaginal delivery intention (CSAOL-2). In total, 37 women delivered vaginally (9.5% of the cohort), including 33 with a documented VBAC-2 attempt and 4 additional spontaneous cases with unknown intention or unplanned vaginal birth. Spontaneous labor incidence: Of 390 women, 153 (39.2%) experienced spontaneous labor, determining VBAC-2 eligibility: − 81/107 VBAC-2 intenders (75.7%) − 68/283 non-intenders (24.0%) + 4 spontaneous vaginal births.

**Table 2:** Intended and final delivery mode

mode of delivery	VBAC-2 outcome	Forceps	ERCS-2	CSAOL-2	CSAOL-2 as immediate emergency cesarean
VBAC-2 intention *n* = 107	16 (15.0%)	17 (15.9%)	26 (24.3%)	48(44.6%) (= fTOLAC-2)	2 (1.9%)
RCS-2 intended *n* = 265	1 (0.4%)	1 (0.4%)	204 (77%)	59 (22.3%)	0
Unknown intention *n* = 18	1 (5.6%)	1 (5.6%)	7 (39%)	9 (50%)	0
Total	**18**	**19**	**237**	**116**	**2**

### VBAC-2: maternal and neonatal outcomes by outcome groups

No instances of complete uterine rupture were observed in the VBAC-2 attempts. Overall complete rupture rate: 1.0% (4/390). Dehiscence occurred in approximately 8.2% of cases (32/390). The mean blood loss was found to be lower in the VBAC-2 group compared to the repeat cesarean section group (371 ml vs. 471 ml; *p* < 0.001). As demonstrated in Table 3, other complications are rare and comparable. Subgroup analyses comparing elective cesarean section with cesarean section after onset of labor (Supplement Table 1 a) or after failed trial of labor after cesarean (fTOLAC-2) reveal no statistically significant differences in maternal outcomes (Supplement Table 2a).

**Table 3:** Maternal outcomes – outcome-defined groups VBAC-2 vs. repeat CS-2: This group included all kind of cesarean sections after two prior cesareans: elective repeat cesarean section (ERCS-2) and cesarean section after onset of labor (CSAOL). Continuous variables are presented as mean ± standard deviation and were compared using Student’s *t*-test or Mann–Whitney *U* test, as appropriate. Categorical variables were compared using Fisher’s exact test

Characteristics	VBAC-2 *n* = 37	RCS-2 *n* = 353	*p*-value
Wound infection	0	4 (1.13%)	0.515
Bladder injury	0	3 (0.85%)	0.574
Postpartum hemorrhage	0	5 (1.42%)	0.466
Blood transfusion	1 (2.7%)	2 (0.57%)	0.157
Hysterectomy	0	0	1
Blood loss (ml, mean ± SD)	371 ± 230	471 ± 230	< 0.001
Perineal injury 3./4.degrees	2 (5.4%)	0	< 0.001

The neonatal outcomes were favorable overall. The pH of the umbilical artery was found to be marginally lower in the VBAC-2 cohort (mean 7.21) when compared to the repeat cesarean section group (7.30; p < 0.001). Although statistically significant, values remained within clinically acceptable ranges. However, no instances of perinatal asphyxia or fatalities were observed. Admissions to the NICU are not a frequent occurrence. Furthermore, the duration of hospitalization was found to be reduced in cases of VBAC-2 (2.3 vs. 4.2 days; p < 0.001) (Table 4). Similarly, subgroup analyses revealed no statistically significant differences in neonatal outcomes (Supplement Table 1b and Table 2b)

**Table 4:** Neonatal outcomes – outcome-defined groups VBAC-2 vs. repeat CS-2

Characteristics	VBAC-2 *n* = 37	RCS-2 *n* = 353	*p*-value
pHa (mean ± SD)	7.21 ± 0.05	7.3 ± 0.06	< 0.0001
pHa < 7,15 (%)	3 (8.11)	5 (1.42)	0.063
Base Excess (mean ± SD)	-5.1 ± 3.28	-0.75 ± 2.6	< 0.0001
Base Excess <-8 (%)	4 (10.8)	9 (2.6)	0.008
APGAR 5’ < 7	0	3 (0.85)	0.574
Intubation (%)	0	1 (0.28)	0.262
Admission to NICU (%)	1 (2.78)	28 (7.93)	1.00
Days at NICU (mean ± SD)	0.1 (0.548)	0.97 (6.33)	0.550
Age at demission (mean ± SD)	2.33 (1.37)	4.24 (5.97)	< 0.0001
Adjustment disorder following the birth of a newborn (%)	0	9 (3.72)	0.291
Perinatal asphyxia	0	0	
Respiratory distress (%)	0	1 (0.42)	0.723
Chorioamnionitis (%)	0	1 (0.42)	0.723

### Cesarean section after labor onset: maternal and neonatal outcomes by outcome groups

Comparison of CSAOL-2 and ERCS-2 groups revealed no significant differences in maternal or neonatal morbidity, estimated blood loss, uterine rupture/dehiscence, or neonatal respiratory morbidity (Tables 5 and 6). Similarly, Supplementary Table S1 a (maternal morbidity: ERCS-2 vs. CSAOL-2, both without vaginal delivery intention) and Supplementary Table S1b (fetal outcomes: ERCS-2 vs. CSAOL-2, both without vaginal delivery intention) demonstrated no significant differences between groups.

**Table 5:** Maternal outcomes – outcome-defined groups elective CS-2 vs. CSAOL-2 (including vaginal delivery intention): This group included all elective repeat cesarean section (ERCS-2) and cesarean section after onset of labor (CSAOL-2) without vaginal delivery intention and with failed TOLAC-2

Characteristics	ERCS-2 *n* = 237	CSAOL-2 *n* = 116	*p*-value
Wound infection (%)	3 (1.27)	1 (0.86)	0.736
Bladder injury (%)	2 (0.84)	1 (0.86)	0.986
Postpartum hemorrhage (%)	4 (1.69)	1 (0.86)	0.538
Blood transfusion (%)	2 (0.84)	0	0.321
Hysterectomy	0	0	
Blood loss (ml, mean ± SD)	468.8 (238)	474.1 (217)	0.747
Complete rupture (%)	1 (0.42)	3 (2.59)	0.071
Uterine dehiscence (%)	19 (8.02)	13 (11.21)	0.327
Total rupture rate (%)	20 (8.44)	16 (13.79)	0.118

**Table 6:** neonatal outcomes – outcome-defined groups elective CS-2 vs. CSAOL-2 (including vaginal delivery intention).

Characteristics	ERCS-2 *n* = 237	CSAOL-2 *n* = 116	*p*-value
pHa (mean ± SD)	7.30 ± 0.056	7.29 ± 0.055	0.102
pHa < 7.15 (%)	5 (2.11)	0	0.115
Base Excess (mean ± SD)	-0.75 ± 2.7	-0.76 ± 2.4	0.657
Base Excess <-8 (%)	6 (2.53)	3 (2.59)	0.976
APGAR 5’ < 7 (%)	2 (0.84)	1 (0.86)	0.986
Intubation (%)	0	1 (0.86)	0.152
Admission to NICU (%)	19 (8.02)	9 (7.76)	0.933
Days at NICU (mean ± SD)	1.35 ± 7.95	0.35 ± 1.63	0.784
Age at demission (mean ± SD)	4.75 ± 7.47	3.45 (1.64)	0.005
Adjustment disorder following the birth of a newborn (%)	8 (5.33)	1 (1.09)	0.090
Perinatal asphyxia	0	0	
Respiratory distress (%)	1 (0.42)	0	0.428
Chorioamnionitis (%)	1 (0.42)	0	0.428
Fetal death	0	0	

Exploratory nominal logistic regression examined associations between selected outcomes and vaginal delivery intention (Table 7). Low umbilical artery pH (< 7.15) was linked to higher odds of intention (OR 8.26, p = 0.011), while other factors showed no significant associations. Due to low event rates, results are descriptive

**Table 7:** exploratory nominal logistic regression – Predictors of vaginal delivery intention

Dependent variable: Intention to deliver vaginally	Odds ratio	95-% CI	*P* value (Wald test)
pH < 7.15	8.26	1.86–57.11	0.011
Fetal admission to NICU	0.71	0.26–1.71	0.478
Postpartum hemmorrhage	0.67	0.03–4.66	0.729
Uterine rupture	1.09	0.48–2.29	0.829

## Discussion

### Main findings

It is estimated that approximately one-third of women intending VBAC-2 successfully achieved vaginal birth (30.8% intent-to-treat). No complete uterine ruptures occurred among VBAC-2 attempts (overall rate: 1.0%), and VBAC-2 showed reduced blood loss versus repeat cesarean (371 vs. 471 ml, *p* < 0.001). CSAOL-2 showed no statistically significant differences in maternal or neonatal morbidity compared with ERCS-2. The 39.2% spontaneous labor rate (153/390) represents a key protocol limitation, with only 75.7% of VBAC-2 intenders becoming eligible. Exploratory nominal logistic regression examined factors associated with *vaginal delivery intention*. Low umbilical artery pH (< 7.15) was linked to higher odds of VBAC-2 intention (OR 8.26, 95% CI 1.86–57.11, *p* = 0.011), possibly reflecting clinical management of higher-risk cases. Other outcomes (NICU admission OR 0.71, postpartum hemorrhage OR 0.67, uterine rupture OR 1.09) showed no significant associations (all *p* > 0.05). Due to low event rates, these results are descriptive only.

### Comparison with literature

Our observation of reduced maternal blood loss with successful VBAC-2 aligns with the findings of Garg et al. [6], who reported more favorable maternal outcomes with vaginal delivery. In line with Macones et al. [7], no significant differences in short-term maternal morbidity were observed between vaginal and elective cesarean delivery, underscoring that VBAC-2 safety depends on careful selection and standardized management. Compared with international reports, success rates in our cohort were lower than the commonly reported 45–60% [8], but consistent with Dombrowski et al. [9]. This likely reflects rigorous candidate selection, avoidance of induction, and a low threshold for operative intervention. The high rate of operative vaginal delivery exceeds that reported in most series [8] and reflects a deliberately conservative strategy to shorten the second stage and mitigate rupture risk, emphasizing the need for transparent counseling regarding the likelihood of instrumental birth. Despite this, rates of high-grade perineal trauma were lower than previously reported (e.g., Perslev et al. [10]), possibly due to differences in patient selection or delivery techniques.

The overall uterine rupture rate of 1.0% in the present cohort is consistent with the published data (1–2%) [7, 11, 12]. Although overall maternal and neonatal morbidity did not differ significantly between ERCS-2 and CSAOL-2, a nonsignificant numerical increase in complete uterine rupture was observed among women undergoing cesarean delivery after labor onset. Given the limited sample size and the rarity of this complication, these findings should be interpreted with caution. This pattern warrants caution and argues against broadly promoting CSAOL-2 as a universally preferable strategy. These findings are consistent with those reported by Tahseen et al. [12] and Dombrowski et al. [9], though they contrast with the results observed by Wagner et al. [13], who reported increased maternal risks after intrapartum cesarean sections. Whilst numerous studies have highlighted elevated risks after failed TOLAC [3, 14], cesarean delivery after spontaneous labor onset in our cohort was not associated with statistically significant excess short-term maternal or neonatal morbidity compared with elective repeat cesarean. However, the absence of demonstrated benefit and the limited statistical power preclude recommending this approach as a routine management strategy. Although neonatal respiratory morbidity and length of hospital stay were numerically lower in the CSAOL-2 group, these differences did not reach statistical significance and should be interpreted cautiously.

### Strengths and limitations

A significant strength of this study is the utilization of a predefined institutional protocol, which ensures consistent management and facilitates meaningful subgroup comparisons. The incorporation of CSAOL-2, explicitly defined and analyzed, provides novel insight, as this option is seldom examined in VBAC-2 cohorts. The institution’s strengths lie in its clear delineation of rupture versus dehiscence and the active involvement of senior personnel in all deliveries.

This study also has several limitations. The retrospective design restricts the ability to draw causal inferences and is susceptible to selection bias and unmeasured confounding factors. The small number of women who attempted a vaginal birth after two previous cesarean sections, and the rarity of several maternal and neonatal outcomes, reduced statistical power and increased the risk of type II error. Primary analyses were therefore bivariate and unadjusted, and exploratory regression analyses were limited by low event rates and small subgroup sizes, which prevented the reliable identification of independent associations.

Although all surgical and delivery reports were systematically reviewed for evidence of full-thickness uterine rupture or partial dehiscence, the retrospective assessment introduces potential documentation bias and observer variability, which may have affected the accuracy of rupture classification. Finally, long-term maternal health, psychological well-being and patient-reported experiences were not considered. These limitations should be considered when interpreting the findings.

### Clinical implications

VBAC-2 can be safely offered to carefully selected women in standardized settings, but counseling should emphasize the modest success rate, the high likelihood of operative vaginal delivery, and the small yet present rupture risk. From a counseling perspective, it is also relevant that more than three quarters of women who intended VBAC-2 (75.7%) did in fact enter spontaneous labor within the expectant management window, which can be used to provide realistic expectations about the likelihood of becoming eligible for TOLAC-2 under similar protocols.

Patient preferences regarding predictability versus cesarean avoidance vary widely. In our cohort, some women chose CSAOL-2 as a patient-driven compromise between VBAC-2 and ERCS-2. While CSAOL-2 did not show excess overall maternal or neonatal morbidity compared with ERCS-2, the observed non-significant trend toward higher complete uterine rupture rates (*p* = 0.071), together with the limited sample size, precludes recommending this approach routinely or presenting it as a standard counseling option.

Counseling should therefore focus primarily on the two established strategies – carefully selected VBAC-2 and elective repeat cesarean (ERCS-2) – with individualized discussion of CSAOL-2 only for women who explicitly remain undecided about mode of delivery despite comprehensive counseling. Prospective multicenter studies are needed to better quantify risks and refine selection criteria.

## Conclusion

Under a strict non‑induction protocol, VBAC‑2 after two prior cesarean sections was feasible but infrequent, with modest success (30.8% intent‑to‑treat, 9.5% overall) and a high rate of instrumental delivery. No complete uterine ruptures occurred among VBAC‑2 attempts in this limited cohort, but the study was not powered to exclude rare events.

From a clinical perspective, elective repeat cesarean (ERCS‑2) and carefully selected VBAC‑2 remain the two principal management strategies for women with two previous cesarean sections. Cesarean after spontaneous labor onset (CSAOL‑2) did not demonstrate clear maternal or neonatal benefit compared with ERCS‑2. This approach should therefore not be routinely recommended and may be considered only as an individualized option for women who remain undecided about their preferred mode of delivery despite thorough counseling.

Counseling should transparently communicate protocol‑specific success rates, the likelihood of spontaneous labor, and the uncertainty regarding rare complications. Prospective multicenter studies are needed to better quantify risks and refine selection criteria.

## Supplementary Information

Supplementary Material 1: Supplementary Table S1 a: Maternal morbidity: ERCS-2 vs. CSAOL-2 without vaginal intention. Supplementary Table S1 b Fetal Outcomes: ERCS-2 vs. CSAOL-2 without vaginal intention. Supplementary Table S2 a: Failed TOLAC-2 vs. ERCS-2 maternal morbidity. Supplementary Table S2 b: Failed TOLAC-2 vs. ERCS-2 fetal outcomes.

Supplementary Material 2

## Data Availability

Data may be made available from the corresponding author upon reasonable request, in accordance with institutional and privacy regulations.

## Abbreviations

VBAC-2: Vaginal Birth After two Cesareans (spontaneous or forceps-assisted)
ERCS-2: Elective Repeat Cesarean before labor
CSAOL-2: Cesarean After Spontaneous Labor onset
Planned CSAOL-2: Planned Cesarean Section after spontaneous Labor Onset
fTOLAC-2: failed Trial of Labor After Cesarean

## References

[1] Betran AP, Ye J, Moller A-B, Souza JP, Zhang J. Trends and projections of cesarean section rates: global and regional estimates. BMJ Glob Health. 2021;6:e005671. 10.1136/bmjgh-2021-005671. 34130991 10.1136/bmjgh-2021-005671 PMC8208001

[2] Deutsche Gesellschaft für Gynäkologie und Geburtshilfe (DGGG). Sectio caesarea AWMF 015–084 Leitlinie S3 Version 1, Stand Juni 2020 n.d.

[3] ACOG Practice Bulletin No. 205: Vaginal Birth After Cesarean Delivery. Obstet Gynecol. 2019;133:e110–27. 10.1097/AOG.0000000000003078. 30681543 10.1097/AOG.0000000000003078

[4] Salim R, Shalev E. Health implications resulting from the timing of elective cesarean delivery. Reprod Biol Endocrinol. 2010;8:68. 10.1186/1477-7827-8-68. 20565934 10.1186/1477-7827-8-68 PMC2902487

[5] Kok N, Wiersma IC, Opmeer BC, De Graaf IM, Mol BW, Pajkrt E. Sonographic measurement of lower uterine segment thickness to predict uterine rupture during a trial of labor in women with previous Cesarean section: a meta-analysis. Ultrasound Obstet Gynecol. 2013;42:132–9. 10.1002/uog.12479. 23576473 10.1002/uog.12479

[6] Garg VK, Ekuma-Nkama EN. Vaginal birth following two cesarean sections. Int J Gynecol Obstet. 2005;88:53–4. 10.1016/j.ijgo.2004.09.009. 10.1016/j.ijgo.2004.09.009 15617707

[7] Macones GA, Cahill A, Pare E, Stamilio DM, Ratcliffe S, Stevens E, et al. Obstetric outcomes in women with two prior cesarean deliveries: Is vaginal birth after cesarean delivery a viable option? Am J Obstet Gynecol. 2005;192:1223–8. 10.1016/j.ajog.2004.12.082. 15846208 10.1016/j.ajog.2004.12.082

[8] De Leo R, La Gamba DA, Manzoni P, De Lorenzi R, Torresan S, Franchi M, et al. Vaginal Birth after Two Previous Cesarean Sections versus Elective Repeated Cesarean: A Retrospective Study. Am J Perinatol. 2020;37:S84–8. 10.1055/s-0040-1714344. 32898889 10.1055/s-0040-1714344

[9] Dombrowski M, Illuzzi JL, Reddy UM, Lipkind HS, Lee HC, Lin H, et al. Trial of Labor After Two Prior Cesarean Deliveries: Patient and Hospital Characteristics and Birth Outcomes. Obstet Gynecol. 2020;136:109–17. 10.1097/AOG.0000000000003845. 32541284 10.1097/AOG.0000000000003845 PMC7321852

[10] Perslev K, Mørch E, Jangö H. Increased risk of obstetric anal sphincter injury in women undergoing vaginal delivery after cesarean section: A systematic review and meta-analysis. BJOG Int J Obstet Gynaecol. 2022;129:1961–8. 10.1111/1471-0528.17227. 10.1111/1471-0528.17227 35596697

[11] Landon MB, Spong CY, Thom E, Hauth JC, Bloom SL, Varner MW, et al. Risk of Uterine Rupture With a Trial of Labor in Women With Multiple and Single Prior Cesarean Delivery. Obstet Gynecol. 2006;108:12–20. 10.1097/01.AOG.0000224694.32531.f3. 16816050 10.1097/01.AOG.0000224694.32531.f3

[12] Tahseen S, Griffiths M. Vaginal birth after two cesarean sections (VBAC-2)-a systematic review with meta-analysis of success rate and adverse outcomes of VBAC-2 versus VBAC-1 and repeat (third) cesarean sections: Success rate & adverse outcomes of vaginal birth after two cesarean sections. BJOG Int J Obstet Gynaecol. 2010;117:5–19. 10.1111/j.1471-0528.2009.02351.x. 10.1111/j.1471-0528.2009.02351.x 19781046

[13] Wagner SM, Bicocca MJ, Mendez-Figueroa H, Gupta M, Reddy UM, Chauhan SP. Neonatal and maternal outcomes with trial of labor after two prior cesarean births: stratified by history of vaginal birth. J Matern Fetal Neonatal Med. 2022;35:6013–20. 10.1080/14767058.2021.1903862. 33792462 10.1080/14767058.2021.1903862

[14] Bretelle F, Cravello L, Shojai R, Roger V, D’ercole C, Blanc B. Vaginal birth following two previous cesarean sections. Eur J Obstet Gynecol Reprod Biol. 2001;94:23–6. 10.1016/S0301-2115(00)00328-6. 11134821 10.1016/s0301-2115(00)00328-6

