# Supplementary material for: Vaginal birth after two cesarean sections (VBAC-2) under a standardized protocol: success rates, safety, and cesarean after spontaneous labor as an alternative
Source: BMC Pregnancy Childbirth. 2026 Mar 12;26:385. doi: 10.1186/s12884-026-08905-9 (PMC13063759; doi:10.1186/s12884-026-08905-9)
Supplement: Supplementary file 1 — Supplementary Material 1: Supplementary Table S1 a: Maternal morbidity: ERCS-2 vs. CSAOL-2 without vaginal intention. Supplementary Table S1 b Fetal Outcomes: ERCS-2 vs. CSAOL-2 without vaginal intention. Supplementary Table S2 a: Failed TOLAC-2 vs. ERCS-2 maternal morbidity. Supplementary Table S2 b: Failed TOLAC-2 vs. ERCS-2 fetal outcomes. [file 12884_2026_8905_MOESM1_ESM.docx]

**Supplementary Material**

- Supplementary Table S1a: Maternal morbidity: ERCS-2 vs. CSAOL-2 without vaginal intention
- Supplementary Table S1b Fetal Outcomes: ERCS-2 vs. CSAOL-2 without vaginal intention
- Supplementary Table S2a: Failed TOLAC-2 vs. ERCS-2 maternal morbidity
- Supplementary Table S2b: Failed TOLAC-2 vs. ERCS-2 fetal outcomes

| Characteristics | ERCS-2  n= 126 | CSAOL-2  n=45 | p-Value |
| --- | --- | --- | --- |
| Wound infection (%) | 2 (1.59) | 1 (2.22) | 1.00 |
| Bladder injury (%) | 1 (0.79) | 1 (2.22) | 0.458 |
| Atonia (%) | 4 (3.17) | 0 | 0.574 |
| Blood transfusion (%) | 3 (2.38) | 0 | 0.567 |
| Hysterectomy | 0 | 0 |  |
| Blood loss (ml, mean ± SD) | 488.09 (305.25) | 445.55 (126.05) | 0.609 |
| Complete rupture (%) | 1 (0.79) | 2 (4.44) | 0.170 |
| Covered rupture (%) | 10 (7.94) | 5 (11.11) | 0.199 |
| Total rupture rate (%) | 11 (8.73) | 7 (15.56) | 0.256 |

Supplementary Table S1a: **Maternal morbidity: ERCS-2 vs. CSAOL-2**

| Characteristics | ERCS-2  n= 126 | CSAOL-2  n=45 | p-Value |
| --- | --- | --- | --- |
| pHa (mean ± SD) | 7.30 (0.05) | 7.30 (0.05) | 0.219 |
| pHa < 7,15 (%) | 2 (1.59) | 0 | 1.00 |
| Base Excess (mean ± SD) | -0.737 (2.40) | -0.678 (1.96) | 0.826 |
| Base Excess <-8 (%) | 1 (0.79) | 0 | 1.00 |
| APGAR 5' <4 | 0 | 0 |  |
| Intubation (%) | 0 | 0 |  |
| Admission to NICU (%) | 8 (6.35) | 3 (6.67) | 1.00 |
| Days at NICU (mean ± SD) | 0.92 (4.631) | 0.288 (1.217) | 1.00 |
| Age at demission (mean ± SD) | 4.34 (4.10) | 3.53 (1.51) | 0.069 |
| Adjustment Disorder following the birth of a newborn (%) | 4 (3.17) | 0 | 0.574 |
| Perinatal asphyxia | 0 | 0 |  |
| Respiratory distress (%) | 0 | 0 |  |
| Amnioninfection (%) | 1 (0.79) | 0 | 1.00 |

Supplementary Table S1b **Fetal Outcomes: ERCS-2 vs. CSAOL-2**

| Characteristics | RCS-2 n= 171 | failed TOLAC2 n= 52 | p-Value |
| --- | --- | --- | --- |
| Wound infection (%) | 3 (1.75) | 1 (1.92) | 1.00 |
| Bladder injury (%) | 2 (1.17) | 0 | 1.00 |
| Atonia (%) | 4 (2.34) | 1 (1.92) | 1.00 |
| Blood transfusion (%) | 3 (1.75) | 0 | 1.00 |
| Hysterectomy | 0 | 0 |  |
| Blood loss (ml, mean ± SD) | 476.9 (270.14) | 479.8 (259.19) | 0.439 |
| Complete rupture (%) | 3 (1.75) | 0 | 1.00 |
| Covered rupture (%) | 15 (8.77) | 7 (13.46) | 0.503 |
| Total rupture rate (%) | 18 (10.53) | 7 (13.46) | 0.616 |

Supplementary Table S2a: Failed TOLAC-2 vs. ERCS-2 maternal morbidity

RCS-2 repeat cesarean section included elective cesarean and cesarean after onset of labor without vaginal intention.

| Characteristics | RCS-2 n= 171 | failed TOLAC2 n= 52 | p-Value |
| --- | --- | --- | --- |
| pHa (mean ± SD) | 7.301 (0.052) | 7.29 (0.076) | 0.733 |
| pHa < 7,15 (%) | 2 (1.17) | 3 (5.77) | 0.084 |
| Base Excess (mean ± SD) | -0,72 (2.29) | -1,3 (2.89) | 0.360 |
| Base Excess <-8 (%) | 1 (0.58) | 1 (1.92) | 0.413 |
| APGAR 5' <4 | 0 | 0 |  |
| Intubation (%) | 0 | 1 (1.92) | 0.233 |
| Admission to NICU (%) | 11 (6.43) | 2 (3.85) | 0.737 |
| Days at NICU (mean ± SD) | 0.76 (4.01) | 0.215 (1.54) | 0.220 |
| Age at demission (mean ± SD) | 4.135 (3.62) | 3.275 (1.57) | 0.010 |
| Adjustment Disorder following the birth of a newborn (%) | 4 (2.34) | 1 (1.96) | 1.00 |
| Perinatal asphyxia | 0 | 0 |  |
| Respiratory distress (%) | 0 | 0 |  |
| Amnioninfection (%) | 1 (0.58) | 0 | 1.00 |
| Fetal death | 0 | 0 |  |

Supplementary Table S2b: Failed TOLAC-2 vs. ERCS-2 fetal outcomes
