# Supplementary material for: Vaginal birth after two cesarean sections (VBAC-2) under a standardized protocol: success rates, safety, and cesarean after spontaneous labor as an alternative
Source: BMC Pregnancy Childbirth. 2026 Mar 12;26:385. doi: 10.1186/s12884-026-08905-9 (PMC13063759; doi:10.1186/s12884-026-08905-9)
Supplement: Supplementary file 2 — Supplementary Material 2 [file 12884_2026_8905_MOESM2_ESM.docx]

**Supplementary Material**

- Supplementary Table S1a: Maternal morbidity: ERCS-2 vs. CSAOL-2 without vaginal intention
- Supplementary Table S1b Fetal Outcomes: ERCS-2 vs. CSAOL-2 without vaginal intention
- Supplementary Table S2a: Failed TOLAC-2 vs. RCS-2 maternal morbidity
- Supplementary Table S2b: Failed TOLAC-2 vs. RCS-2 fetal outcomes

| Characteristics | ERCS-2  n= 211 | CSAOL-2  n=68 | p-Value |
| --- | --- | --- | --- |
| Wound infection (%) | 2 (0.95) | 1 (1.47) | 0.716 |
| Bladder injury (%) | 2 (0.95) | 1 (1.47) | 0.716 |
| Postpartum hemmorhage (%) | 4 (1.90) | 0 | 0.253 |
| Blood transfusion (%) | 2 (0.95) | 0 | 0.424 |
| Hysterectomy | 0 | 0 |  |
| Blood loss (ml, mean ± SD) | 474.0 ± 250 | 446.3 ± 128 | 0.547 |
| Complete rupture (%) | 1 (0.47) | 3 (4.41) | 0.018 |
| Uterine dehiscence (%) | 15 (7.11) | 7 (10.29) | 0.397 |
| Total rupture rate (%) | 16 (7.58) | 10 (14.71) | 0.079 |

Supplementary Table S1a: **Maternal morbidity: ERCS-2 vs. CSAOL-2**

| Characteristics | ERCS-2  n= 211 | CSAOL-2  n=68 | p-Value |
| --- | --- | --- | --- |
| pHa (mean ± SD) | 7.30 ± 0.05 | 7.30 ± 0.05 | 0.135 |
| pHa < 7,15 (%) | 2 (0.95) | 0 | 0.420 |
| Base Excess (mean ± SD) | -0.732 ± 2.6 | -0.731 ± 1.8 | 0.645 |
| Base Excess <-8 (%) | 6 (2.84) | 1 (1.47) | 0.529 |
| APGAR 5' < 7 (%) | 2 (0.95) | 0 | 0.420 |
| Intubation (%) | 0 | 0 |  |
| Admission to NICU (%) | 17 (8.06) | 6 (8.82) | 0.842 |
| Days at NICU (mean ± SD) | 1.51 ± 8.4 | 0.38 ± 1.5 | 0.992 |

Supplementary Table S1b **Fetal Outcomes: ERCS-2 vs. CSAOL-2**

| Characteristics | RCS-2 n= 307 | failed TOLAC2 n= 48 | p-Value |
| --- | --- | --- | --- |
| Wound infection (%) | 4 (1.30) | 0 | 0.436 |
| Bladder injury (%) | 3 (0.98) | 0 | 0.359 |
| Postpartum hemorrhage (%) | 4 (1.30) | 1 (2.17) | 0.641 |
| Blood transfusion (%) | 2 (0.65) | 0 | 0.583 |
| Hysterectomy | 0 | 0 |  |
| Blood loss (ml, mean ± SD) | 463 ± 217 | 520 ± 302 | 0.652 |
| Complete rupture (%) | 4 (1.30) | 0 | 0.436 |
| Uterine dehiscence (%) | 26 (8.5) | 6 (13.0) | 0.314 |
| Total rupture rate (%) | 30 (9.8) | 6 (13.0) | 0.494 |

Supplementary Table S2a: Failed TOLAC-2 vs. RCS-2 maternal morbidity

RCS-2 repeat cesarean section included elective cesarean and cesarean after onset of labor without vaginal intention.

| Characteristics | RCS-2 n= 307 | failed TOLAC2 n= 48 | p-Value |
| --- | --- | --- | --- |
| Arterial pH mean ± SD | 7.3 ± 0.05 | 7.29 ± 0.07 | 0.423 |
| Arterial pH < 7.15 (%) | 5 (1.63) | 0 | 0.383 |
| Base excess mean ± SD | -0.76 ± 2.52 | -0.72 ± 2.98 | 0.956 |
| BE < - 8 mmol/l (%) | 7 (2.28) | 2 (4.35) | 0.407 |
| 5 min APGAR < 7 | 3 (0.98) | 0 | 0.501 |
| Intubation (%) | 1 (0.33) | 0 | 0.687 |
| Admission to NICU (%) | 26 (8.47) | 2 (4.35) | 0.335 |

Supplementary Table S2b: Failed TOLAC-2 vs. RCS-2 fetal outcomes
